# Dysregulation of JAK-STAT pathway in hematological malignancies and JAK inhibitors for clinical application

**DOI:** 10.1186/2050-7771-1-5

**Published:** 2013-01-16

**Authors:** Muhammad Furqan, Nikhil Mukhi, Byung Lee, Delong Liu

**Affiliations:** 1 Department of Medicine, New York Medical College and Westchester Medical Center, Valhalla, NY, 10595, USA; 2 California Cancer Center, 7257 N. Fresno St., Fresno, CA, 93720-2950, USA

## Abstract

JAK-STAT (Janus associated kinase-signal transducer and activator of transcription) pathway plays a critical role in transduction of extracellular signals from cytokines and growth factors involved in hematopoiesis, immune regulation, fertility, lactation, growth and embryogenesis. JAK family contains four cytoplasmic tyrosine kinases, JAK1-3 and Tyk2. Seven STAT proteins have been identified in human cells, STAT1-6, including STAT5a and STAT5b. Negative regulators of JAK–STAT pathways include tyrosine phosphatases (SHP1 and 2, CD45), protein inhibitors of activated STATs (PIAS), suppressors of cytokine signaling (SOCS) proteins, and cytokine-inducible SH2-containing protein (CIS). Dysregulation of JAK-STAT pathway have been found to be key events in a variety of hematological malignancies. JAK inhibitors are among the first successful agents reaching clinical application. Ruxolitinib (Jakafi), a non-selective inhibitor of JAK1 & 2, has been approved by FDA for patients with intermediate to high risk primary or secondary myelofibrosis. This review will also summarize early data on selective JAK inhibitors, including SAR302503 (TG101348), lestaurtinib (CEP701), CYT387, SB1518 (pacritinib), LY2784544, XL019, BMS-911543, NS-018, and AZD1480.

## Introduction

JAK-STAT (Janus associated kinase-signal transducer and activator of transcription) pathway is one of the critical intracellular signaling cascades in transduction of extracellular signals to the nucleus to control gene expression. A variety of cytokines and growth factors complete their physiological tasks through JAK-STAT pathway, including hematopoiesis, immune-regulation, fertility, lactation, growth and embryogenesis [1-6].

**Figure 1:**
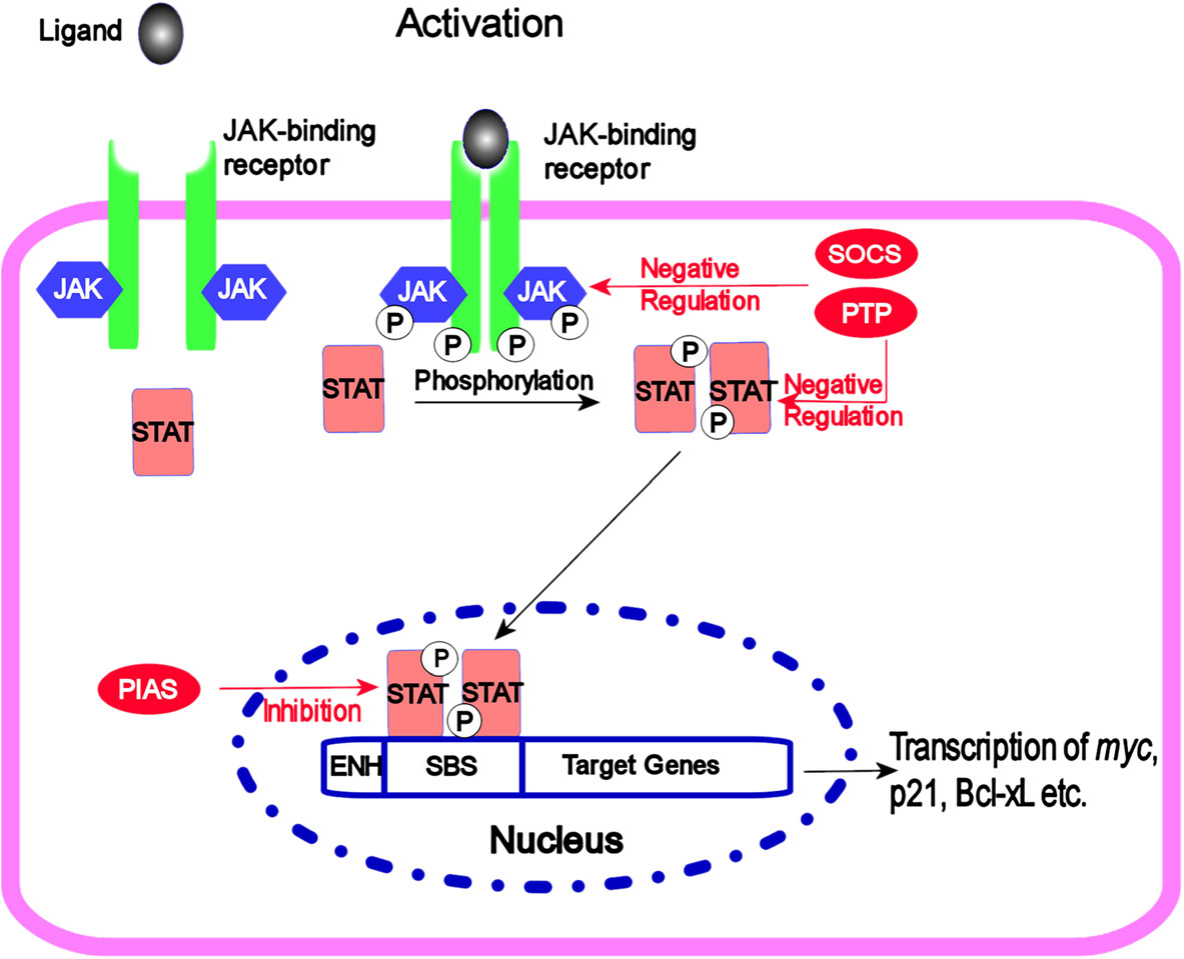
**Signaling cascade of JAK-STAT pathway.** Binding of the ligand to cytokine receptor induces receptor dimerization and activation of receptor associated JAK kinase, which in turn phosphorylates STAT proteins. After forming a homodimer, STAT proteins translocate to the nucleus to control gene expression. Negative regulation of the JAK-STAT pathway is provided by phospho-tyrosine phosphatases (PTP), member for SOCS family proteins and PIAS proteins (shown in red).

JAK family contains four cytoplasmic tyrosine kinases, JAK1-3 and Tyk2 [7]. These kinases bind to the juxta-membrane region of cytokine receptors [8]. Each molecule contains seven JAK homology domains (JH1-7). The carboxyl JH1 domain contains the catalytic activity, whereas N-terminal JH7 domain is responsible for receptor binding. JH2 domain has significant homology to JH1 but lacks enzymatic activity and therefore is a pseudo-kinase domain. Binding of a ligand to its receptor results in receptor dimerization, leading to activation of the JAK kinase activity. Subsequently activated JAKs phosphorylate cytoplasmic domain of the receptor [9]. Activated JAK-cytokine receptor complex recruits and phosphorylates specific cytoplasmic transcription factors called STAT proteins [10]. Seven STAT proteins have been identified in human cells, STAT1-6, including STAT5a and STAT5b [7]. Phosphorylation of specific STAT proteins results in their dimerization and subsequent translocation into the nucleus to interact with various regulatory elements for gene expression (Figure 1) [11,12].

## Regulation of JAK-STAT pathway

Three major mechanisms have been implicated to negatively regulate the JAK-STAT pathway [11]. Figure 1 depicts the positive and negative regulation of the JAK-STAT pathway.

### Tyrosine phosphatases

Src homology-2 (SH2) containing tyrosine phosphatase and CD45 tyrosine phosphatase play a major role in modulating JAK-STAT pathway. SH2 containing tyrosine phosphatases include SHP1 and SHP2 (shatterproof 1 & 2). Their SH2 domains allow attachment to the phospho-tyrosine residues present on activated receptors, JAKs or STAT proteins, leading to dephosphorylation of the substrates. SHP1 is mainly expressed in hematopoietic cells, epithelial and smooth muscle cells whereas SHP2 is ubiquitous. The expression of SHP1 gene was found to be silenced by promoter hypermethylation in various hematological malignancies [10]. CD45 transmembrane tyrosine phosphatase is the second type of tyrosine phosphatase that negatively regulates JAK-STAT pathway. CD45, the leukocyte common antigen, is expressed in hematopoietic cells. It has two phosphatase domains in its intracellular region, though only one is active. Mice deficient for CD45 show hyper-activation of JAK1 and JAK3 [10].

### Protein inhibitors of activated STATs (PIAS)

PIAS family consists of PIAS1, PIAS3, PIASx and PIASy. Biochemical assays have shown that PIAS3 and PIASx interact with STAT3 and STAT4 respectively, while PIAS1 and PIASy with STAT1 [13,14]. PIAS1 and PIAS3 exert negative regulation by blocking the DNA binding of STAT1 and STAT3, respectively [15,16]. On the other hand, PIASx and PIASy repress the transcriptional activity of STAT1 and STAT4 by recruiting co-repressor molecules such as histone deacetylases [17].

### Suppressors of cytokine signaling (SOCS) proteins

The suppressor of cytokine signaling (SOCS) family is composed of SOCS-1 to SOCS-7 and cytokine-inducible SH2-containing protein (CIS). SOCS proteins are cytokine-induced negative feedback-loop regulators of JAK-STAT signaling. Each member protein in this family has a central SH2 domain, variable amino-terminal domain and a carboxy-terminal of 40 amino acids module known as SOCS box. The central SH2 domain directs the target binding of each CIS/SOCS protein [18]. Three mechanisms have been proposed by which a SOCS protein provides negative modulation. First, SOCS binds the phospho-tyrosine residues on the receptors and physically blocks the STATs from binding to its receptors. Second, SOCS proteins can bind directly to its specific JAKs or to the receptors and inhibit the corresponding JAK kinase activity. Third, SOCS associate with the elongin BC complex and cullin 2, accelerating the ubiquination of JAKs and presumably the receptors [19].

Endosomal degradation of JAK/receptor complex through receptor mediated endocytosis appears to be another mechanism involved in the regulation of the pathway. STAT5ß dimerizes with the full-length, wild-type protein, STAT5α, upon activation. This leads to negative regulation of the JAK-STAT signaling cascade in addition to the major mechanisms described above [20].

## JAK-STAT pathway and hematological malignancies

Dysregulation in JAK-STAT pathway have been described in a variety of hematological malignancies, especially in myeloid disorders. These events may play key roles in initiation and progression of respective diseases.

## Myeloproliferative Neoplasms (MPN)

### Ph negative MPNs

Short interfering RNA and JAK2 inhibitor (AG490) have been used to study the activity of JAK2. JAK2 was shown to be central to the erythropoietin-independent erythroid proliferation in polycythemia vera (PV). A common mutation (V617F) in the JH2 pseudo-kinase domain of JAK2 was shown in 80% of the patients [21]. This results in constitutive activation of STAT5 and a malignant phenotype [22]. Other investigators confirmed the fact and showed that 30-50% of patients with essential thrombocythemia (ET) and primary myelofibrosis (PMF) also harbor the same mutation [23-25].

Allelic burden of JAK2V617F was found to play an important role in determining a distinct clinico-pathologic phenotype from the same mutation [22,26]. It is suggested by the fact that homozygous JAK2V617F mutation is 30% in PV compared to 2-4% in ET [22,26]. Transgenic mouse models and clinical cases support the gene-dosage model for MPN pathogenesis. It was observed that high allele burden leads to PV phenotype, whereas reduction in allelic burden leads to ET [22,27-29].

In patients with PV who are negative for JAK2V617F, Scott *et al.,* used a candidate gene approach to search for alleles that can activate JAK signaling. Their work demonstrated at least 8 distinct mutations involving residues 538 to 543 in exon 12 region of JAK2 [29].

Irrespective of JAK2V617F status, patients with PV exhibits high STAT3 and 5 activities, patients with ET have high STAT3 activity, whereas patients with PMF showed low activities of both STAT3 and 5. It is evident that preferential activation of JAK2–STAT5 or JAK2–STAT3 signaling determines PV, ET and PMF phenotype. However patients with JAK2V617F-negative ET or PMF may harbor mutations in other genes in JAK–STAT pathway [30].

### Atypical MPNs

Rare JAK2 gene rearrangement can result in atypical form of chronic myeloid leukemia (aCML). These gene rearrangements include PCM1-JAK2, TEL-JAK2 and BCR-JAK2 [31-33]. Rarely, JAK2V617F mutation can present phenotypically as chronic myelomonocytic leukemia /aCML.

PCM1-JAK2 gene rearrangement was discovered in the translocation, t(8;9)(p22;p24). It is reported in few cases with atypical CML, chronic eosinophilic leukemia, AML (M2, M6), pre B-ALL and T-cell lymphoma.

### Lymphoma and lymphoid leukemia

#### T-cell lymphoma and leukemia

JAK1 gene plays a unique role in lymphohematopoiesis. Flex *et al.,* showed several acquired JAK1 missense mutations in adult lymphoblastic leukemia, especially in T-cell precursor type where it accounted for 18% of the cases. It was associated with advanced age and poor prognosis [34-36].

Translocation between short-arm of chromosome 9 (harboring JAK2 gene) and 12, t(9;12)(p24;p13) results in the formation of a fusion protein, TEL-JAK2. This results in constitutive JAK2 activation. It was found in a case of childhood T-cell acute lymphoblastic leukemia [37] and in a case with pre-B-cell ALL [33].

#### Adult T-cell Leukemia (HTLV-1 positive)

Human T-cell lymphotropic virus-1 encoded Tax protein suppresses apoptosis through constitutive activation of the NFκB pathway. This is sufficient for immortalization of CD4^+^ lymphocytes. HTLV-1 infected T-cells over-express IL-2, IL-15 as well as IL-2 and IL-15 receptors by Tax-induced NFκB activation. These in turn activate JAK3-STAT5 pathway leading to lymphocyte proliferation and adult T-cell lymphoma /leukemia [38].

#### T-cell Large Granular Lymphocytic Leukemia, Anaplastic Large cell Lymphoma, Angioimmunoblastic Lymphoma and Sezary Syndrome

Dysregulation of STAT proteins also contributes to the pathogenesis of various types of lymphoid malignancies. Schade *et al.,* reported increase activity of STAT3 in T-cell large granular lymphocytic leukemia [39]. Constitutive activation of STAT3 & 5 was also found to be an important event in the pathogenesis of anaplastic large cell lymphoma, T-cell angioimmunoblastic lymphoma and Sezary syndrome [40,41].

## B-Cell lymphoma and leukemia

### Acute lymphoblastic leukemia

Kearney *et al.,* performed sequencing of exon 14 of JAK2 gene in patients with Down syndrome associated ALL. An acquired somatic point mutation invovling residue R683 was found in 28% of cases [42]. A different mutation in JAK2 gene in a patient with Down syndrome associated precursor B-cell lymphoblastic leukemia was reported by Malinge *et al.,* This mutation was in-frame deletion of five-amino acid residues from position 682 to 686, named as IREED mutation, in the pseudokinase domain of JAK2. This IREED mutant remains constitutively active and induces cytokine independence in Ba/F3 cells [43].

Another JAK2 mutation involves fusion with SSBP2 in t(5;9)(q14.1;p24.1) translocation. It was detected in a patient with pre B-cell ALL [44].

### Chronic lymphocytic leukemia

Constitutive activation of STAT1 & 3 was noticed in transformed B-cells in patients with chronic lymphocytic leukemia (CLL). Activated STAT1 & 3 proteins in malignant B-cells were phosporylated at serine instead of tyrosine residue. It remained uncertain whether activated STAT involved directly in the pathophysiology of this disease or represented a pure epi-phenomenon [45].

### Primary mediastinal B-cell lymphoma

A bi-allelic mutation in SOCS-1 has been reported in primary mediastinal B-cell lymphoma. This results in delayed turnover of phosphorylated JAK2 and increased activity of STAT5, which may explain the uncontrolled growth of malignant cells [46].

### Hodgkin lymphoma

Gene rearrangement by translocation involving JAK2, t(4;9)(q21;p24), has been reported in 2 cases of classic Hodgkin lymphoma by a group from Belgium [47]. This gene re-arrangement creates a novel fusion product, SEC31A-JAK2. This oncogenic SEC31A-JAK2 has constitutively active tyrosine kinase activity. Mutations of SOCS-1 have also been identified in Hodgkin lymphoma [48].

## Myeloid leukemia

### Acute myeloid leukemia

Increased activity of STAT3 has been reported in 20-50% of AML patients while STAT5 and STAT1 activation is uncommon [49,50]. The pathologic value of activated STAT3 in AML is unclear. It was also found in acute promyelocytic leukemia (APL). An interstitial deletion within chromosome 17 has been described to result in STAT5b-RARα fusion protein. This was implied to serve as a key pathogenic event in a patient with APL [51]. Transformation from severe congenital neutropenia due to truncated G-CSF receptor to AML also involves dysregulation of STAT proteins. Truncated G-CSF receptor lacks a crucial binding site for SOCS-3, thus relieving inhibition of STAT5, but not of STAT3. This favors myeloid proliferation over differentiation. These truncated receptors are also resistant to down modulation through ubiquination [52].

Rare point mutations, one in JAK2 (T875N) and three in JAK3 (A572V, V722I, P132T), have been described in patients with acute megakaryocytic luekemia [52]. PCM1-JAK2 gene rearrangement mentioned above was found in M2 and M6 AML [36].

## Myelodysplastic syndrome (MDS)

Perturbation in JAK-STAT pathway is rare in MDS. Only 4.2% patients with MDS were found to harbor JAK2V617F mutation in a large screening series [53]. Of interest is a disease entity called refractory anemia with ringed sideroblast associated with marked thrombocytosis (RARS-t). These patients have features of both MDS and MPN. It was recently shown that 6 out of 9 (67%) patients with RARS-t have JAK2V617F mutation [54].

## Multiple myeloma

Constitutive expression of STAT3 has been seen frequently in patients with multiple myeloma and lymphoma [55]. However, overexpression of anti-apoptotic proteins of Bcl-2, Bcl-XL and Mcl-1 may not rely on STAT3 activation. The role of this pathway in pathogenesis of myeloma remains to be determined [56].

## Novel inhibitors of JAK-STAT pathway

The specific and profound involvement of JAK-STAT pathway in various hematological malignancies, especially Bcr-Abl negative MPNs, makes them ideal targets for pharmacological intervention [57]. Several strategies have been proposed to inhibit or modulate this pathway [58,59]. JAK inhibitors are among the first successful agents reaching clinical application [60,61].

## JAK inhibitors

There are at least 10 different JAK inhibitors undergoing various phases of clinical trials (Table 1). Ruxolitinib (Jakafi manufactured by Incyte, Inc) has been approved by FDA for patients with intermediate- to high- risk primary or secondary myelofibrosis.

**Table 1:** JAK inhibitors in clinical trials

**Drugs**	**JAK Target**	**Non-JAK Target**	**Trials: status**	**Disease under study**
**(Ref)**				
INCB018424 (Ruxolitinib)	JAK1 & 2	NR	III: Reported	PMF, Post ET/PV MF,
			III: recruiting	PV (intolerant or resistant to HU)
FDA Approved			II: Recruiting	PV, ET
[62-67]			I/II: Recruiting	AML/ALL (Relapse/Refractory)
			II: Completed	MM (Relapse/Refractory)
			II: Recruiting	DLBCL/T-Cell Lymphoma
			II: Active	Advanced Hem Malignancies
SAR302503	JAK2	FLT3	III: Recruiting	PMF
(TG101348)		RET	II: Recruiting	PV, ET
[70]				
CEP-701	JAK2	FLT3	II: Completed	ET, PV
(Lestaurtinib)	JAK3	VEGFR2	II: Completed	AML (FLT3)
[71,72]		TRKA	III: Recruiting	ALL (children)
		RET	II: Recruiting	PMF
CYT387	JAK1,2	PKD3, PRKD1, CDK2, ROCK2, JNK1	II: Recruiting	PMF, Post PV/ET MF
[77,78]	Tyk2			
SB1518	JAK2	FLT3	II: Completed	PMF
[83,84]			II: Completed	Adv Myeloid malignancies
			II: Completed	Adv Lymphoid malignancies
			II: Recruiting	MDS
LY2784544	JAK2V617F	NA	I/II: Recruiting	PMF, ET, PV
[85]				
XL019	JAK2	NA	I: Terminated	PMF
[86]				
AZD1480	JAK1-3	FGFR1, FLT4, ARK5, ALK4, Aurora-A	I/II: Active	PMF, Post ET/PV MF
[91]				
BMS-911543	JAK2	No information	I/II: active	PMF
[90]				
NS018	JAK2	Src Kinases	I/II: active	PMF, Post ET/PV MF
[89]				

Ref, references; NR, none reported; HU, hydroxyurea; AML, acute myeloid leukemia; ALL, acute lymphoid leukemia; MM, multiple Myeloma; DLBCL, diffuse large B-cell lymphoma.

### Ruxolitinib

Ruxolitinib (INCB018424) is a non-selective JAK1 & 2 inhibitor. Multiple phase I/II studies in various hematological malignancies are underway. Among these, results are most mature for PMF and post –ET /PV myelofibrosis.

### Phase I/II studies

A total of 153 patients with myelofibrosis were recruited in a phase I/II study. Dose limiting toxicity was thrombocytopenia with maximum tolerated dose of 25 mg twice daily. Effect on JAK2V617F allele burden or bone marrow fibrosis was insignificant [62]. After a longer follow up, it was described that abrupt discontinuation can lead to serious adverse events like acute relapse of constitutional symptoms, rapid and painful enlargement of spleen and shock. By the time of last report on the follow up of these patients 35% of patient died and 10% transformed to leukemia [63,64].

Eghtedar *et al.,* reported a phase I/II study of ruxolitinib in patients with relapsed /refractory AML. This study has shown 7% complete remission rate. It was noticed that patients who achieved complete remission were those with post-MPN AML [65].

### Phase III studies

Ruxolitinib has undergone 2 separate randomized controlled trials, referred to as the COntrolled MyeloFibrosis study with ORal JAK inhibitor Treatment (COMFORT) I & II (Table 2) [66,67]. Patients were randomized between ruxolitinib vs placebo (COMFORT I) or best available therapy (COMFORT II). Patients with PMF or post –ET /PV myelofibrosis must have high or intermediate international prognostic scores, palpable spleen ≥5 cm below the left costal margin, and platelet count equal or greater than 100 × 10^9^ /L. Patients were recruited irrespective of JAK2 gene mutation status. Differences in the design of these trials and major outcomes are summarized in Table 2.

**Table 2:** COMFORT-I & II trials on ruxolitinib

**Methodology**	**COMFORT-I**	**COMFORT-II**
Design	1:1 randomization, vs. placebo	2:1 randomization, vs. best available therapy
Number of patients	155 (ruxolitib) vs. 154 (placebo)	146 (ruxolitinib) vs. 73 (BAT)
Dose of ruxolitinib	15-20 mg twice daily based on platelet count	15-20 mg twice daily based on platelet count
Primary end point	reduction in spleen size > 35% at 24 week	reduction of spleen size > 35% at 48 week
Results		
35% reduction in spleen size	42% vs. 0.7% at 24 week	28% vs. 0% at 48 week
Duration of spleen response *	67% patient maintained the spleen response for 48 weeks	80% maintained the response at 12 month follow up
Major Adverse events:	Anemia (45%), Thrombocytopenia (13%), headache, pyrexia	Anemia, Diarrhea, Arthralgia, fatigue, abdominal pain
Discontinuation Rate	11% vs. 10.6%	8% vs. 5%

BAT, best available therapy; * , secondary end-points ; QOL, quality of life ; ns, non significant.

These trials support the palliative role of ruxolitinib which significantly reduced spleen volume and constitutional symptoms. The responses were not specific for JAK2 mutation.

### SAR302503 (TG101348)

SAR302503 is a selective JAK2 inhibitor with activity on both wild type and mutant JAK2 [68,69]. Fifty nine patients were enrolled in a multi-center phase I/II trial with high-or- intermediate-risk primary or post-PV/ET myelofibrosis. Patients with platelet count equal or greater than 50 x 10^9^ /L were eligible. Reversible increase in serum amylase was the dose limiting toxicity. Serious adverse events included mainly myelosuppression and gastrointestinal toxicity. A spleen response of 47% was seen after one year of treatment. JAK2V617F allele burden was significantly decreased at 6 months in mutation-positive patients (n = 51; P = .04). The response was higher in patients with allele burden greater than 20% (n = 23; P < .01) [70]. A multinational, randomized, placebo controlled phase III trial has been launched recently for patients with high risk myelofibrosis.

### Lestaurtinib (CEP701)

Lestaurtinib is an orally available indolocarbazole derivative. It was originally identified as an inhibitor of the neurotropin receptor TrkA. It was found to also inhibit FLT3, JAK2 and JAK3. Due to its strong activity against FLT3, it is under investigation in patients with acute leukemia. A phase I/II study of lestaurtinib was done in 22 patients with JAK2-mutated myelofibrosis. The patients received 80 mg twice-daily. The response rate was 27%. The median duration of response was more than 14 months. However, bone marrow fibrosis or JAK2V617F allele burden was not significantly altered [71].

In another phase 2 study, lestaurtinib was given to 39 high-risk patients with JAK2V617F-positive PV (n=27) or ET (n=12). After 18 weeks of treatment, a significant decrease in spleen size was reported in 83% of the patients. Six patient developed thrombosis. The increase in the thrombotic events remains a concern for this agent [72].

### CYT387

CYT387 is an ATP-competitive small molecule JAK1/JAK2 inhibitor [73-76]. It has an IC50 of 11 and 18 nM for JAK1 and JAK2, respectively. Unlike lestaurtinib, CYT387 has significantly less activity against other kinases, including JAK3 (IC50=0.16 μM).

One hundred sixty three patients with high- or intermediate- risk myelofibrosis were enrolled in a phase I/II trial of CYT387. The median duration of treatment was 6.6 months. Dose limiting toxicities included hyper-lipasemia and headaches. Maximum tolerated dose was 300 mg/day. The response rates for reduction of spleen size, anemia, and constitutional symptom were 45%, 50%, and more than 50%, respectively. It is interesting to note that responses were also seen in patients who had previously failed ruxolitinib or SAR302503. Surprisingly, 58% of transfusion-dependent patients became transfusion-independent. Transient light headedness and hypotension were the common adverse events. Treatment-related severe anemia was infrequent (< 1%). This property separates it from ruxolitinib and SAR302503 [77,78].

### SB1518 (pacritinib)

Pacritinib is another orally bioavailable ATP-competitive small molecule inhibitor of JAK2 and the mutant JAK2V617F [79-82]. It inhibitis JAK-STAT signaling pathway through caspase-dependent apoptosis. In a phase I/II trial, 34 patients with primary or post PV/ET myelofibrosis were enrolled. The median time on study was 8.2 months. Patients with low blood counts were not excluded. Most common adverse events were diarrhea, nausea and vomiting. The primary end point was spleen response, which was 32%. The median duration of spleen response has not been reached at the recent update [83].

In another phase I trial, 30 patients with relapsed/refractory lymphoma were enrolled. The dose was escalated from 200–600 mg/day. Severe adverse events included cytopenia, internal hemorrhage, various infections and abdominal distention. No patient achieved complete remission. Three patients had partial remission and 13 patients had stable disease [84]. Phase II trial is ongoing.

### LY2784544

LY2784544 is a selective mutant JAK2 kinase inhibitor with an IC50 of 68 nM. This molecule selectively inhibited the JAK2 V617F-STAT5 signaling at a concentration that was 41-fold lower than that needed for the wild type (WT) JAK2 signaling (IC50 = 55 nM for V617F vs. 2260 nM for WT ) [60]. A phase I/II trial recruited 19 patients with Ph negative MPN. Maximum tolerated dose was 120 mg/day. Spleen response was 22% for patients with MF. JAK2V617F allele burden was not significantly decreased. Main adverse events included diarrhea, nausea, anemia and electrolyte imbalance [85].

### XL019

XL019 represents another oral selective JAK2 inhibitor (IC50= 2nM for JAK2, 130 nM for JAK1, 250 nM for JAK3, 340 nM for TYK2) [86,87]. A phase I study recruited 21 patients with myelofibrosis. 16 patients had JAK2V617F, one had MPL W515F. Dose ranged from 25 to 300 mg. Surprisingly, there was no hematological adverse events. Improvement in clinical outcome observed in all patients with JAK2V617F or MPL activating mutations [86]. Despite these results, further development of clinical trials using XL019 was aborted out of the concerns for neurologic toxicities [88].

### NS-018, BMS-911543 and AZD1480

These JAK inhibitors are also selective JAK inhibitors at very early stage of drug development [48,89-91]. NS-018 was shown to be highly selective against JAK2 with IC50 <1 nM [89]. It had 30–50 fold greater selectivity for JAK2 over other JAK-family kinases, such as JAK1, JAK3 and Tyk2. BMS-911543 is a functionally selective JAK2 inhibitor [90]. AZD1480 has been shown both in vitro and in vivo to inhibit the growth of multiple malignancies by inhibiting phosphorylation of JAK2, STAT3 and MAPK signaling proteins [91-99]. Clinical investigations on these agents are underway.

## Conclusion and future directions

Currently the benefits of therapy with JAK inhibitors in MPNs are palliative in nature. This is suggested by inability of ruxolitinib to influence the natural history of the diseases and by the fact that the responses are unrelated to JAK mutation status. JAK2V617F mutation may be a key event but not necessarily a primary factor driving the tumorigenesis of MPNs. Other molecular events may precede the acquisition of JAK2V617F mutation in these disorders. Novel selective JAK2 inhibitors are the active focus for further clinical investigations [60,61,88].

In addition to JAK inhibitors, targeting STAT signaling is emerging as an attractive approach to inhibit tumorigenesis. Various strategies have been employed to influence STAT signaling at multiple levels, though these approaches are still at early stage and only supported by pre-clinical data [13,100-107]. Further advancement in understanding the molecular events and refining the targets can potentially lead to further clinical benefit. Simultaneous intervention at multiple levels of the signaling cascade by combining different targeting agents may offer additional advantages and is worthwhile studying.

## Competing interest

The authors have no relevant conflicts of interest.

## Authors’ contributions

All authors have contributed to data preparation, drafting and revising the manuscripts. All authors have read and approved the final manuscript.
